# Dietary *Piper sarmentosum* Roxb. Extract Improves Antioxidant Capacity, Lipid Metabolism and Flavor Formation in Male Hainan Black Goat Kids Under Heat Stress

**DOI:** 10.3390/antiox15060721

**Published:** 2026-06-05

**Authors:** Guodong Ren, Ziyang Sheng, Yixin Chen, Tingshuo Yang, Nan Zhang, Renlong Lv, Hanlin Zhou, Hailing Luo

**Affiliations:** 1 Sanya Institute of China Agricultural University, Sanya 572025, China; guodongren630@cau.edu.cn (G.R.); sheng17552733897@163.com (Z.S.); chenyixin260507@163.com (Y.C.); yangtingshuo@cau.edu.cn (T.Y.); z12yzhangnan@163.com (N.Z.); 2 State Key Laboratory of Animal Nutrition and Feeding, College of Animal Science and Technology, China Agricultural University, No. 2 Yuanmingyuan West Road, Haidian, Beijing 100193, China; 3 Tropical Crops Genetic Resources Institute, Chinese Academy of Tropical Agricultural Sciences, No. 4 Xueyuan Road, Haikou 571101, China; lvrenlong@catas.cn (R.L.); zhouhanlin@catas.cn (H.Z.); 4 Zhanjiang Experimental Station, Chinese Academy of Tropical Agricultural Sciences, Zhanjiang 524013, China

**Keywords:** heat stress, *Piper sarmentosum* Roxb. extract, Hainan black goat, free fatty acids, flavoromics, transcriptomics

## Abstract

Global warming-induced heat stress causes oxidative imbalance and reduced productivity in livestock. This study investigated the effects of dietary supplementation with *Piper sarmentosum* Roxb. extract (PSE) on antioxidant capacity, lipid metabolism, and flavor formation in goats under heat stress. Thirty-six healthy 3-month-old male Hainan black goat kids were fed a basal diet supplemented with 0, 200, 400, or 600 mg/kg PSE (dry-matter basis) for 105 days. Specifically, PSE significantly enhanced antioxidant capacity, as indicated by increased total antioxidant capacity and glutathione peroxidase activity, along with reduced malondialdehyde levels (*p* < 0.05). These changes were accompanied by a decrease in n-6 polyunsaturated free fatty acids and a relative increase in saturated fatty acids (*p* < 0.05), suggesting a potential improvement in lipid oxidative stability. Further flavoromics analysis revealed a marked shift in meat volatile profiles, characterized by increased esters associated with fruity and waxy notes and decreased aldehydes and alcohols contributing to green and herbal odors. Muscle transcriptomic results further indicated enrichment of redox-related pathways, including oxidoreductase activity and reactive oxygen species metabolism. Overall, PSE, particularly at 600 mg/kg, enhanced antioxidant capacity and regulated redox status and lipid metabolism under heat stress, potentially contributing to improved meat oxidative stability and altered flavor compound formation.

## 1. Introduction

Tropical regions have historically dominated global livestock agriculture, both in terms of animal numbers and total production; in these regions, goats play a crucial role in providing animal-derived protein and supporting income [1]. However, tropical livestock systems face persistent and intensifying environmental constraints. Rising global temperatures and the increasing frequency of heat stress events have been shown to impair animal performance and product quality [2,3,4,5]. Oxidative stress induced by heat stress can significantly compromise the health and productivity of animals, ultimately decreasing meat quality and shelf life [6,7]. Accumulating evidence suggests that oxidative stress under heat stress accelerates lipid peroxidation, leading to the oxidative degradation of polyunsaturated fatty acids and the excessive generation of reactive aldehydes [8], which are closely associated with undesirable meat flavors and reduced product stability. Therefore, lipid peroxidation plays a pivotal role in the formation of volatile flavor compounds, thereby linking oxidative status to meat sensory quality.

The Hainan black goat is a prized indigenous Chinese breed, recognized for its tender texture and desirable flavor sensory characteristics, and represents the primary source of fresh lamb meat in Hainan Province, China [9,10]. However, this breed is predominantly raised under tropical and subtropical climatic conditions, where high heat stress exposure and intense solar radiation may profoundly influence muscle metabolic status through oxidative stress. At present, meat from Hainan black goats is largely obtained under traditional nutritional conditions, with limited application of functional components aimed at modulating oxidative status [11]. Given the economic and cultural importance of Hainan black goat meat, particularly in tropical regions, approaches that relieve heat stress and improve meat quality are of considerable interest. In this context, natural antioxidants extracted from plants and traditional medicinal herbs have drawn attention for their potential benefits to be added to livestock diets to improve meat quality by inhibiting or reducing oxidative deterioration [12,13], while supporting the utilization of native available botanical resources.

*Piper sarmentosum* Roxb. is a medicinal and food homology plant native to Hainan Province and the southeastern coastal regions of China, and it is widely distributed across Southeast Asia [14,15]. As a traditional plant for medicine and food homology, it is traditionally used for treating coughs, gastrointestinal disorders, fever, and related ailments [16]. The remarkable pharmacological activity of *Piper sarmentosum* Roxb. is attributed to its diverse array of bioactive compounds, including essential oils, alkaloids, and flavonoids, which exhibit strong antimicrobial, antioxidant, and anti-inflammatory activities [16,17]. Similarly, *Piper sarmentosum* Roxb. extracts (PSE) exhibit high antioxidant activity [18] and have been shown to modulate inflammatory cytokines through free radical scavenging and potential antioxidant mechanisms [14,18]. Despite these promising bioactivities, the role of PSE as a plant-derived feed additive with potential antioxidative effects in tropical ruminants under heat stress and the integrative mechanisms linking oxidative stress, lipid metabolism remodeling, and flavor formation in goat meat remain largely unclear.

Considering that oxidative stress is one of the key factors contributing to lipid peroxidation under heat stress conditions, we hypothesized that dietary supplementation with PSE could enhance the endogenous antioxidant defense system, potentially mitigating lipid peroxidation and influencing muscle fatty acid composition and flavor compounds. Therefore, this study aimed to evaluate the effects of PSE supplementation on antioxidant capacity, serum biochemical and immune indices, muscle free fatty acid profiles, volatile flavor compounds, and the expression of related genes in Hainan black goats under heat stress. This study also aimed to provide novel insights into the antioxidant-mediated regulation of meat flavor formation and to establish a mechanistic framework linking oxidative stress, lipid metabolism, and flavor development in ruminants.

## 2. Materials and Methods

### 2.1. Animals and Experimental Design

The experimental protocols for the Hainan black goats were approved by the China Agricultural University Animal Care and Use Committee, and the animal feeding and slaughter procedures followed the university’s guidelines (Beijing, China; approval No. AW20206202-1-12).

#### 2.1.1. Preparation of *Piper sarmentosum* Roxb. Extract

The PSE was supplied by the Tropical Crops Genetic Resources Institute Chinese Academy of Tropical Agricultural Sciences. The collection (leaves and stems) and pulverization were according to a previous study [19]. The *Piper sarmentosum* Roxb. powder was extracted with 95% ethanol and recovered by rotary evaporation to obtain *Piper sarmentosum* Roxb. crude extract by the cold leaching method. The crude extract was freeze-dried, mixed with zeolite powder at a 1:1 ratio, and stored in dark bottles at 4 °C until use. Liquid chromatography (Thermo Vanquish UHPLC, Thermo Fisher Scientific, Dreieich, Germany)–tandem mass spectrometry (Q-Exactive HF, Thermo Fisher Scientific, Dreieich, Germany) and gas chromatography (6890N, Agilent Technologies Co., Ltd., Shanghai, China)–tandem mass spectrometry (G5795B, Agilent Technologies Co., Ltd., Shanghai, China) were employed to characterize bioactive compounds and essential oils in the PSE. The detailed analytical procedures are provided in Supplementary Text S1. The content of bioactive compounds in PSE was supplemented as described in Supplementary Table S1, and the flavonoid content (such as vitexin-rhamnoside and vitexin) accounted for 27.5%. The main chemical component of the essential oil of PSE was myristicin (42.48%, Table S2).

#### 2.1.2. Feeding Management and Diet Composition

This study was conducted at the Hainan Black Goat Breeding Base in Danzhou city (109°51′ E, 19°52′ N), Hainan Province, China. According to a single-factor experimental design, thirty-six 3-month-old male Hainan black goat kids (9.53 ± 1.59 kg) were randomly allocated into four groups (*n* = 9 per treatment group). The treatment groups were fed the same basal diet supplemented with 0 (CON), 200, 400, or 600 mg/kg PSE (dry-matter basis) (Supplementary Table S3). The daily dose of PSE for each goat was thoroughly mixed with a small amount of concentrate supplement and administered prior to the morning feeding to ensure complete consumption of the extract. The ingredients and composition of the basal diet are described in Table 1. The basic diet consisted of concentrates (such as corn and soybean meal) and roughage (king grass), in a 50:50 ratio. Each goat was housed individually and fed twice daily at 08:30 h and 15:00 h, with ad libitum access to the same basal diet (dry-matter basis) and fresh water throughout the experimental period. The experimental period was 105 days, with 15 days for adaptation and 90 days for data collection. Prior to commencing the experiment, each steel single-pen was disinfected, and all goats were uniformly dewormed and immunized. A high-precision electronic temperature and humidity recorder (DLX-XXGY05, DELIXI Co., Ltd., Shanghai, China) was used to monitor the daily dry-bulb temperature (DBT) and relative humidity (RH) in the goat house. The temperature–humidity index (THI) was calculated as THI = 0.8 × DBT + RH/100 × (DBT − 14.4) + 46.4, according to Thom [20].

### 2.2. Blood Samples and Biochemical Analysis

#### 2.2.1. Blood Serum Collection

On day 90, prior to the morning feeding, blood samples were collected from the jugular vein of all goats using 10 mL vacuum blood collection tubes (Andermed Co., Ltd., Zibo, China). Blood serum was separated by centrifuging samples at 2000× *g* for 15 min (Centrifuge 5810 R, Eppendorf SE, Hamburg, Germany) and then frozen at −80 °C for further analysis.

#### 2.2.2. Analysis of Blood Serum Parameters

The total protein, albumin, glucose, triglycerides, total cholesterol, high-density lipoprotein, low-density lipoprotein, blood urea nitrogen, creatinine, total antioxidant capacity (T-AOC), superoxide dismutase (SOD), glutathione peroxidase (GSH-Px), and malondialdehyde (MDA) were analyzed using a biochemical analyzer (BioTek Instruments, Winooski, VT, USA) with Nanjing Jiancheng Biochemical Reagent Kits (Nanjing Jiancheng Biochemical Reagent, Nanjing, China). All procedures were conducted following the manufacturer’s instructions. Blood serum TNF-α, IL-1β, IL-2, IL-4, IL-6, IL-10, IgM, IgA and IgG were analyzed using the corresponding enzyme-linked immunosorbent assay kits and a Rayto RT-6100 microplate reader (Rayto Life Sciences Co., Ltd., Shenzhen, China).

### 2.3. Slaughter Procedure and Longissimus lumborum Muscle Collection

#### 2.3.1. Slaughter Procedure

After the feeding trial, all goats were slaughtered after being deprived of feed and water for 12 h. Each goat was weighed prior to slaughter, and the recorded value was defined as the pre-slaughter live weight. Goats were slaughtered following standard procedures described in previous studies [21]. Prior to slaughter, animals were handled carefully to minimize stress. Goats were slaughtered by trained personnel by exsanguination according to standard slaughter procedures and in accordance with protocols approved by the China Agricultural University Animal Care and Use Committee. The hide was removed, followed by separation of the head at the atlanto-occipital articulation and removal of the fore- and hind-limbs at the carpal-metacarpal and tarsal-metatarsal joints, respectively. The weight of organs was recorded immediately after slaughter.

#### 2.3.2. The Organ Weight and *Longissimus lumborum* Muscle Collection

The relative organ weight (organ development index) was calculated as follows: [organ weight (g)/pre-slaughter live weight (kg)] [22]. The meat samples taken from the 6th to 12th ribs of the *Longissimus lumborum* muscle were trimmed of fat. From each goat, all collected samples were immediately placed in liquid nitrogen for further analysis.

### 2.4. Analysis of Free Fatty Acids in Goat Meat

Appropriate amounts of *Longissimus lumborum* samples were transferred into 2 mL Eppendorf tubes and mixed with 500 μL of extraction solvent (isopropanol:n-hexane, 2:3, *v*/*v*) containing 0.2 mg/L internal standard (stearic acid-d35; Sigma-Aldrich Trading Co., Ltd., Shanghai, China) and a 49 MIX of fatty acids standard mixture (ANPEL Laboratory Technologies Inc., Shanghai, China). The mixtures were thoroughly vortexed, homogenized using a ball mill at 40 Hz, and subsequently ultrasonicated in an ice-water bath. Following centrifugation at 12,000 rpm for 15 min at 4 °C, the supernatants were collected. The remaining residues were re-extracted with an additional 500 μL of extraction solvent under identical conditions. Supernatants obtained from the two extraction procedures were combined, and an 800 μL aliquot was transferred to a vacuum freeze concentrator and evaporated to dryness. Subsequently, 500 μL of methanol/trimethylsilyldiazomethane solution (1:2, *v*/*v*) was added, followed by incubation at room temperature for 30 min. The samples were then evaporated to dryness under a gentle nitrogen stream, reconstituted in 160 μL of n-hexane and centrifuged at 12,000 rpm for 1 min. Finally, the resulting supernatants were transferred into sample vials for gas chromatography–mass spectrometry (GC–MS) analysis.

GC–MS analysis was conducted using an Agilent 7890B gas chromatograph coupled to an Agilent 5977B mass spectrometer (Agilent Technologies Co., Ltd., Santa Clara, CA, USA). Separation was achieved using a DB-FastFAME capillary column (90 m × 250 μm × 0.25 μm, Agilent Technologies Co., Ltd., Santa Clara, CA, USA). A 1 μL aliquot of each sample was injected in split mode at a ratio of 5:1. Helium served as the carrier gas, with a pre-injection purge flow rate of 3 mL/min. The oven temperature program was initiated at 50 °C and maintained for 1 min, followed by an increase to 200 °C at 50 °C/min with a 15 min hold. The temperature was then raised to 210 °C at 2 °C/min and maintained for 1 min, followed by a final increase to 230 °C at 10 °C/min and held for 15 min. The temperatures of the injector, quadrupole, and ion source were set at 240 °C, 230 °C, and 150 °C, respectively. Electron impact ionization was performed at −70 eV. Mass spectral data were acquired in scan/SIM mode over an *m*/*z* range of 33–400. For quality control (QC) during sample analysis, one QC standard sample (at concentrations of 0.5, 1.0, and 1.5 mg/L) was inserted for every 10 experimental samples. The measured QC concentrations were used to evaluate the accuracy of the calibration curve and instrument stability. Experiments were considered valid if the QC recovery rate ranged between 80% and 120% and the relative standard deviation (RSD) of the QC measurements was ≤20%.

### 2.5. Flavoromics Analysis in Goat Meat

An appropriate amount of each goat muscle sample (5 g) was accurately weighed, cryogenically ground after liquid nitrogen freezing, and transferred into a 20 mL airtight headspace vial. Then, 10 μL of n-hexyl-d13 solution (1 mg/L), used as the internal standard, was introduced into the sample vial. The mixture samples were incubated at 80 °C for a duration of 10 min. The volatile flavor compounds were extracted from headspace bottles using a solid-phase microextraction (SPME) fiber (divinylbenzene/carboxen/polydimethylsiloxane, 50/30 μm × 1 cm; Supelco, Bellefonte, PA, USA). Before each adsorption, the SPME fiber head was aged at 270 °C for 10 min. The aged SPME fiber adsorbed the sample for 40 min, after which it was desorbed in the inlet for 5 min.

In this study, a comprehensive two-dimensional gas chromatography/time-of-flight mass spectrometry (GC × GC–TOF-MS) analysis was conducted using standard analytical parameters similar to those reported in previous studies [23]. Volatile flavor compounds analysis was carried out using a Pegasus^®^ 4D instrument (LECO; St. Joseph, MI, USA) equipped with an Agilent 8890A GC system (Agilent Technologies, Palo Alto, CA, USA). The device incorporates a dual-stage cryogenic modulator and is connected to a ToFMS detector (LECO). The separation system had a two-dimensional column as follows: the first column (1D) to be chromatographed was a DB-Heavy Wax (30 m × 250 μm × 0.5 μm, Agilent Technologies, Palo Alto, CA, USA), and the second detected chromatographic column (2D) was a Rxi-5Sil MS (2 m× 150 μm × 0.15 μm, Restek, Bellefonte, PA, USA). The carrier gas was high-purity helium (>99.999%) at a constant flow rate of 1.0 mL/min. The GC injector was set to a final value of 250 °C. The initial temperature of the chromatographic chamber was set at 50 °C for a heating period of 2 min. Subsequent to this, the temperature was increased to 230 °C at a rate of 5 °C/min for a period of 5 min. The heating protocol applied to the 2D column exceeded that of the 1D column by 5 °C; yet, it was consistently lower than that of the modulator by 15 °C. The operation of the mass spectrometer was conducted in the EI mode at 70 eV with a range of *m*/*z* 35–550 and a detector voltage setting of 1960 V. A QC sample was included after every six samples. Repeatability was assessed using the RSD of the internal standard, which was ≤10%. Recovery rates for representative spiked standards ranged from 80% to 115%, indicating acceptable accuracy and precision of the analytical procedure. A comparative analysis of volatile flavor compounds was conducted using mass spectra with ≥80% similarity and a retention index (RI) > 600 against the NIST 2020 spectral library (https://webbook.nist.gov/chemistry/, accessed on 12 March 2026, NIST, Gaithersburg, MD, USA). Retention indices were verified against library values (Lib_RI), with an allowable deviation of ΔRI < 20 units.

After normalization with internal standards, the raw data were subjected to an annotation process for the purpose of identifying flavor compounds. This annotation was performed using Chroma ToF search software (version 5.0), which was integrated with the NIST2020 database. The identified compounds were further classified and analyzed using the PubChem database (https://pubchem.ncbi.nlm.nih.gov/, accessed on 12 March 2026) and ClassyFire database to determine the number, relative content, and classification of flavor compounds. The FlavorDB database (https://cosylab.iiitd.edu.in/flavordb/, accessed on 12 March 2026) was applied in the analysis and comparison of the sensory odors arising from the flavor compounds. Orthogonal partial least squares discriminant analysis (OPLS-DA) was conducted to perform dimensionality reduction of volatile flavor compounds using SIMCA-P (version 13.0) software and the ropls package in R (version 3.5.1). For identifying differential volatile flavor compounds (DVOCs), the cutoff criteria were set as follows: a *t*-test *p*-value is less than 0.05, the variable importance in projection (VIP) is greater than 1, and the fold change (FC) is greater than or less than 1. The relative odor activity value (ROAV) method was employed to evaluate volatile flavor compounds [24].

### 2.6. Transcriptomics Analysis in Goat Meat

*Longissimus lumborum* muscle samples stored in liquid nitrogen were used for total RNA isolation. Total RNA was extracted using the TRIzol Reagent (Invitrogen, Carlsbad, CA, USA) following the manufacturer’s instructions. RNA integrity and quality were evaluated using an Agilent 2100 Bioanalyzer (Agilent Technologies, Palo Alto, CA, USA) and further verified by RNase-free agarose gel electrophoresis. Subsequently, eukaryotic mRNA was selectively enriched using Oligo(dT) magnetic beads. The purified mRNA was then fragmented into short sequences with fragmentation buffer and reverse-transcribed into complementary DNA (cDNA) using the NEBNext Ultra RNA Library Prep Kit for Illumina (New England Biolabs, Ipswich, MA, USA). The resulting double-stranded cDNA was subjected to end repair, adenylation at the 3′ ends, and ligation with Illumina sequencing adapters. Adapter-ligated fragments were purified using AMPure XP Beads (1.0× ratio) and subsequently amplified by polymerase chain reaction (PCR) to construct the final sequencing library. The libraries were sequenced on an Illumina NovaSeq 6000 platform by Gene Denovo Biotechnology Co., Ltd. (Guangzhou, China).

Raw sequencing reads obtained from the platform were initially processed using fastp (v0.18.0) to remove adapters and low-quality sequences [25]. To eliminate ribosomal RNA contamination, the filtered reads were aligned against an rRNA database using Bowtie2 (v2.2.8) [26], and unmapped reads were retained as clean reads for subsequent analyses. Clean reads were then aligned to the *Capra hircus* reference genome (Ensembl release 109) using HISAT2 (v2.4) [27]. The resulting alignments for each sample were assembled into transcripts with StringTie (v1.3.1). Gene expression levels were quantified as transcripts per kilobase of exon model per million mapped reads (TPM) using RSEM [28]. Differential expression analysis between groups was conducted using DESeq2 [29]. Genes or transcripts with a *p*-value < 0.05 and an absolute fold change ≥ 2 were considered differentially expressed (DEGs/DETs). Identified DEGs were subsequently subjected to gene ontology pathway enrichment analysis, with *p*-value ≤ 0.05 set as the significance threshold. Bioinformatic analysis was performed using Omicsmart (https://www.omicsmart.com, accessed on 13 April 2026).

### 2.7. Statistical Analysis

Data on goat pre-slaughter live weight, relative organ weight (organ development index), blood serum biochemical parameters, and free fatty acids were analyzed using Proc Mixed Procedure of SAS (SAS^®^ OnDemand for Academics, version 3.82), with four treatments (CON, 200PSE, 400PSE, and 600PSE) as fixed effects and goat included as a random effect to account for individual biological variability. An orthogonal polynomial SAS CONTRAST statement was used to test the linear and quadratic effects of four PES levels. Differences between the means were tested using Tukey’s multiple range test when a significant difference was observed at a 5% critical value. Statistical significance was set at *p* < 0.05, with trends noted at 0.05 ≤ *p* ≤ 0.10. Spearman’s correlation analysis was performed using the Chiplot tool (https://www.chiplot.online/, accessed on 20 April 2026).

## 3. Results

### 3.1. Environmental Factors and Temperature–Humidity Index, Goat Organ Development and Blood Biochemical Parameters

**Figure 1:**
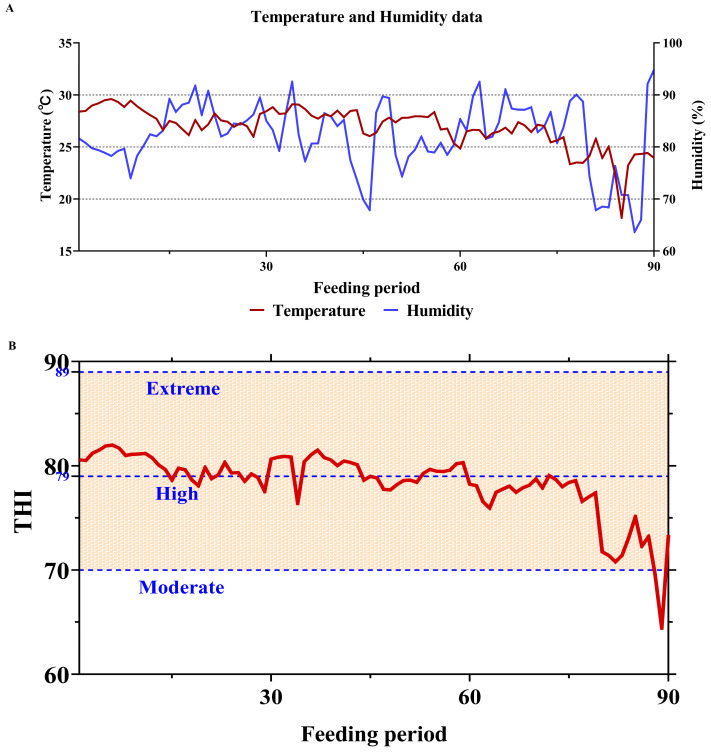
Environmental factors and temperature–humidity index. (**A**) Temperature and humidity statistics. (**B**) Average temperature–humidity index (THI) during the feeding study period. THI values between 70 and 79 are considered moderate heat stress, between 79 and 89 are considered high heat stress, and above 89 are considered extreme heat stress.

The environmental conditions are presented in Figure 1A; they show consistently high ambient dry-bulb temperature and relative humidity throughout the experimental period. Based on previous classifications of the temperature–humidity index (THI), values of 70–79 indicate moderate heat stress, values of 79–89 high heat stress, and values above 89 extreme heat stress [30,31]. As shown in Figure 1B, most THI values during the feeding period exceeded 70, indicating that goats were generally exposed to moderate rather than extreme heat stress conditions. Although some brief fluctuations were observed at the end of the feeding period, THI values predominantly remained within or near the moderate heat stress range.

As shown in Table 2, dietary supplementation with PSE linearly increased the absolute weight of the kidney in goats (*p* < 0.05), whereas no changes were observed in the other organ development indices and pre-slaughter live weight (*p* > 0.05).

**Figure 2:**
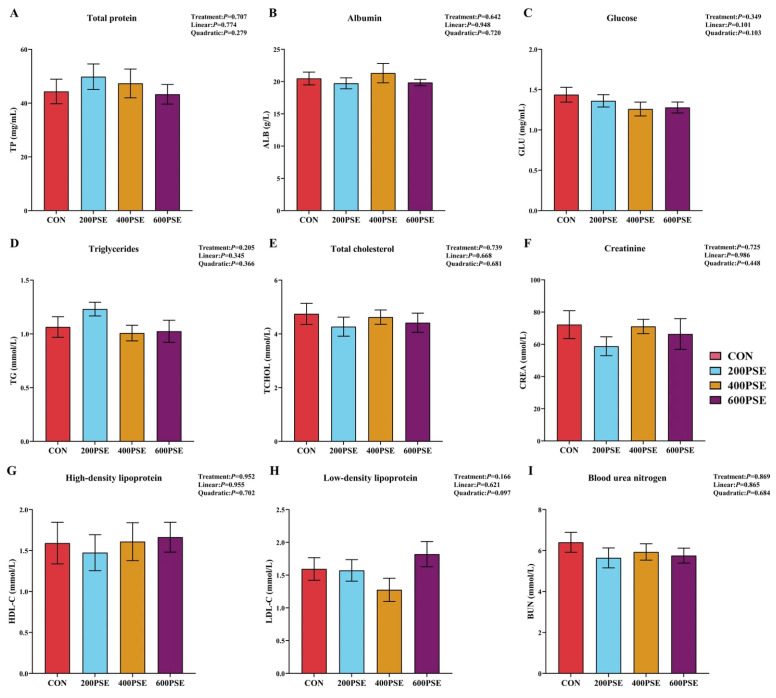
Effect of dietary supplementation with PSE on the blood biochemical parameters of male Hainan black goat kids (*n* = 9 per treatment group). (**A**) Total protein content; (**B**) albumin content; (**C**) glucose content; (**D**) triglycerides content; (**E**) total cholesterol content; (**F**) high-density lipoprotein content; (**G**) low-density lipoprotein content; (**H**) blood urea nitrogen content; (**I**) creatinine content.

**Figure 3:**
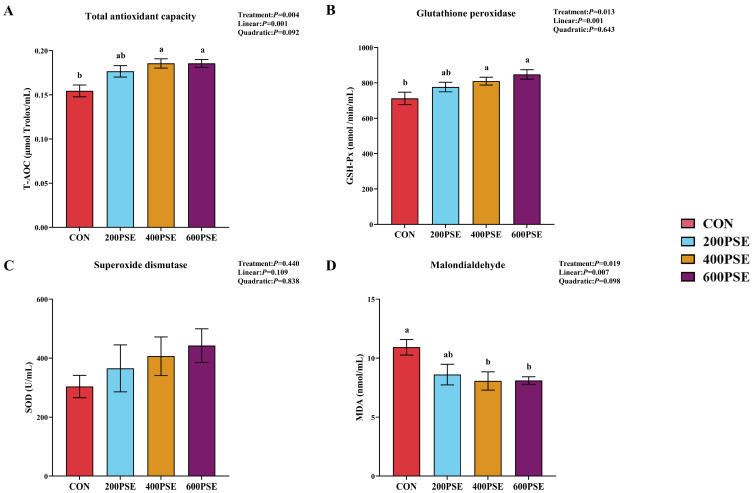
Effect of dietary supplementation with PSE on the antioxidant capacity of male Hainan black goat kids (*n* = 9 per treatment). (**A**) Total antioxidant capacity; (**B**) glutathione peroxidase content; (**C**) superoxide dismutase content; (**D**) malondialdehyde content. Different lowercase letters (a, b) indicate significant differences among treatments (*p* < 0.05).

**Figure 4:**
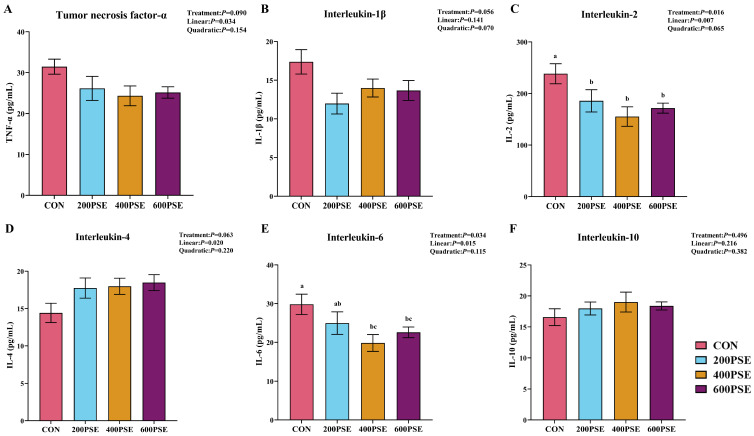

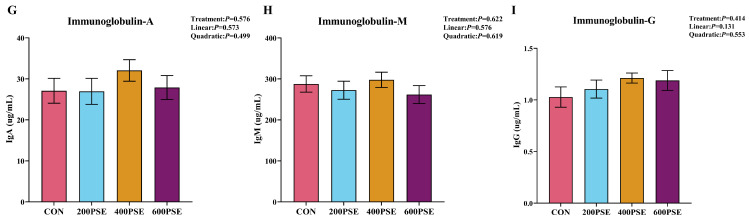
Effect of dietary supplementation with PSE on the blood immune indices of male Hainan black goat kids (*n* = 9 per treatment). (**A**) Tumor necrosis factor-α content; (**B**) interleukin-1β content; (**C**) interleukin-2 content; (**D**) interleukin-4 content; (**E**) interleukin-6 content; (**F**) interleukin-10 content; (**G**) immunoglobulin-A content; (**H**) immunoglobulin-M content; (**I**) immunoglobulin-G content. Different lowercase letters (a–c) indicate significant differences among treatments (*p* < 0.05).

No significant differences were observed in serum biochemical parameters among treatments (*p* > 0.05, Figure 2). Moreover, dietary 400 or 600 mg/kg PSE linearly increased T-AOC (*p* < 0.05) and GSH-Px levels (*p* < 0.05), but decreased MDA (*p* < 0.05, Figure 3). Dietary PSE linearly decreased the blood levels of IL-2 (*p* < 0.05) and IL-6 (*p* < 0.05, Figure 4). Additionally, PSE tended to increase blood IL-4 content (*p* = 0.063) and decrease blood IL-1β and TNF-α levels (*p* = 0.056; *p* = 0.090).

### 3.2. Differences in Free Fatty Acids in Goat Meat

**Figure 5:**
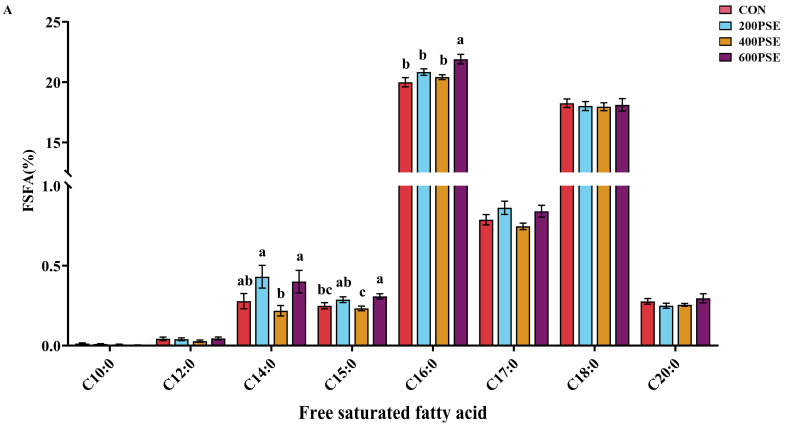

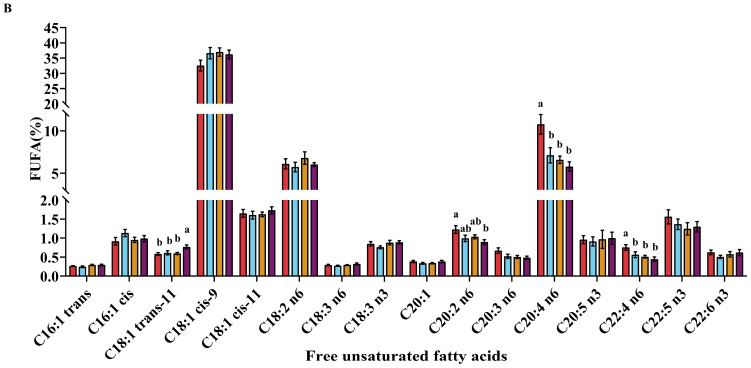
Effect of dietary supplementation with PSE on free fatty acids composition (wet fresh matter basis) of male Hainan black goat kids (*n* = 9 per treatment group). (**A**) Free saturated fatty acids of goat meat; (**B**) Free unsaturated fatty acids of goat meat. Different lowercase letters (a, b, and c) indicate significant differences among treatments (*p* < 0.05).

As shown in Figure 5, compared with the CON group, the concentration of C14:0, C15:0, and C16:0 was increased by dietary supplementation with PSE (*p* < 0.05). Meanwhile, C20:2 n6, C20:4 n6, and C22:4 n6 were decreased by dietary supplementation with PSE (*p* < 0.05), especially at the 600 mg/kg level.

### 3.3. Volatile Flavor Compounds Analysis in Goat Meat

**Figure 6:**
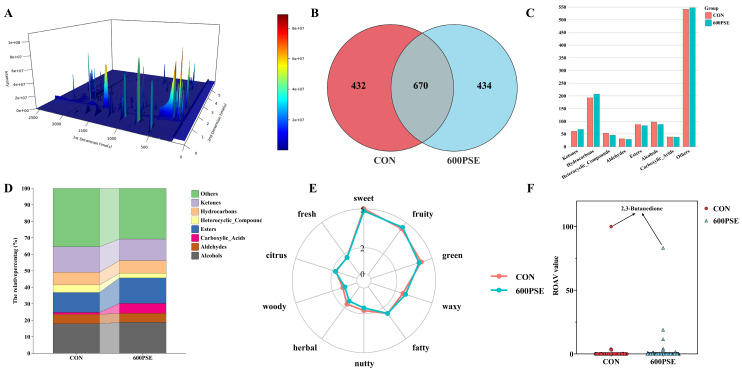
VOC profiles of Hainan black goat meat in CON and 600PSE groups. (**A**) Chromatographic 3D VOC plots of Hainan black goat meat identified by GC×GC-TOF MS. (**B**) The number of identified VOCs in CON and 600PSE groups. (**C**) The distribution of VOCs in CON and 600PSE groups. (**D**) The relative abundance of different VOC categories in CON and 600PSE groups. (**E**) Radar chart of sensory flavor characteristics in CON and 600PSE groups. (**F**) Scatter plot diagram based on the ROAV of VOCs in CON and 600PSE groups.

Given that fatty acids are key precursors of volatile flavor compounds, the observed changes in free fatty acid profiles may underlie the alterations in volatile flavor composition. Significant divergence in free fatty acid composition justifies focusing on the CON and 600PSE groups in order to explore the differences in flavor formation resulting from dietary supplementation with PSE under heat stress. Based on GC×GC-TOF MS analysis, a total of 1536 flavor compounds were identified using the Chemical Abstracts Service (CAS) database (https://www.cas.org/cas-data/cas-registry). As shown in Figure 6B, 434 and 432 specific flavor compounds were observed in the CON and 600PSE groups, respectively. After classification (Figure 6C, Supplementary Table S4), the identified volatile flavor compounds were categorized into eight groups: alcohols, aldehydes, carboxylic acids, esters, heterocyclic compounds, hydrocarbons, ketones, and other unclassified flavor substances. Some volatile flavor compounds remained unclassified. It was observed that alcohols, esters, and ketones were the dominant volatile flavor categories in both CON and 600PSE (Figure 6C,D). The content of carboxylic acids and esters in the 600PSE group was higher than that in the CON group, whereas the content of alcohols and aldehydes was lower than that in the CON group.

According to the analysis using the FlavorDB database, volatile flavor compounds were matched and annotated to determine the sensory flavor characteristics of all goat meat samples. As shown in Figure 6E, both CON and 600PSE goat meat exhibited similar sensory flavor characteristics, with sweet, fruity, and green as the dominant sensory flavor profiles. Compared with the CON group, 600PSE goat meat exhibited stronger fruity and waxy profiles. The sweet, green, and herbal sensory flavor profiles in 600PSE were weaker than those in the CON group. Through the ROAV method, 98 volatile flavor compounds were clearly identified in the volatile sensory threshold analysis. Among these compounds, 2,3-butanedione was identified as a key volatile flavor compound in the muscle of Hainan black goats (Figure 6F and Supplementary Table S5).

### 3.4. Analysis and Difference of Volatile Flavor Compounds in Goat Meat

**Figure 7:**
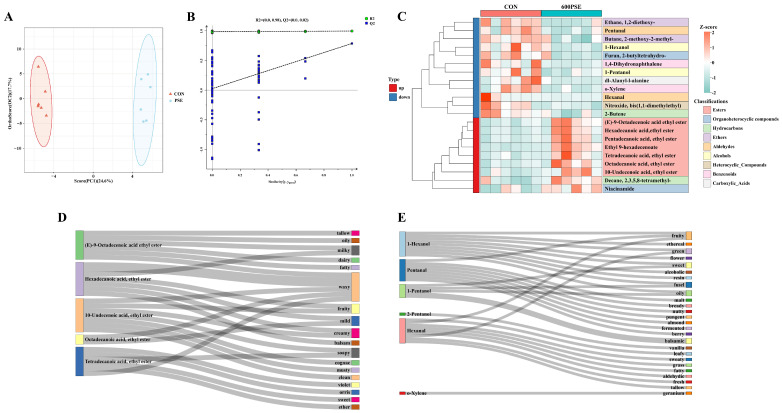
Differential volatile flavor compounds in the CON and 600PSE groups. (**A**) The OPLS-DA score plots are based on DVOCs data. (**B**) The permutation test of OPLS-DA between the CON and 600PSE groups. (**C**) The heatmap of DVOCs between the CON and 600PSE groups. (**D**) The Sankey plot from increased DVOCs to flavor sensory profile between the CON and 600PSE groups. (**E**) The Sankey plot from decreased DVOCs to flavor sensory profile between the CON and 600PSE groups.

After removing missing values (>50%) and replacing the remaining missing values with half of the minimum positive value of each feature, OPLS-DA was used to analyze the differences in volatile flavor compounds between CON and 600PSE goat meat. As shown in Figure 7A, a clear distinction in volatile flavor compositions was observed between the two groups. Under the permutation test for the OPLS-DA models (Q2 = 0.79), all leftmost blue Q2 points were lower than the rightmost original blue Q2 points (Figure 7B), indicating that the OPLS-DA model predictions were reliable and not affected by overfitting.

**Figure 8:**
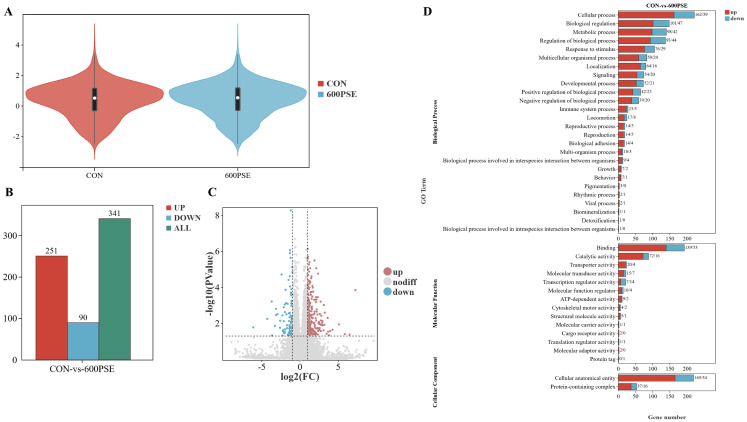
Differential transcriptomics between CON and 600PSE group. (**A**) Violin plots are based on transcriptomics s data; (**B**) The plot of the number of DEGs between CON and 600PSE group; (**C**) The Volcano plot based on DEGs between CON and 600PSE group; (**D**) The pathway plot of DEGs enriched in GO database between CON and 600PSE group.

**Figure 9:**
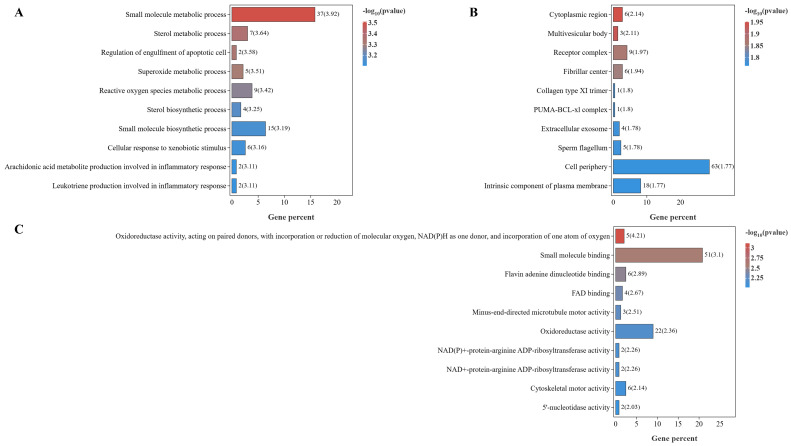
Differential GO enrichment based on DEGs between CON and 600PSE groups. (**A**) The top 10 differential GO enrichment pathways in biological processes. (**B**) The top 10 differential GO enrichment pathways in cellular components. (**C**) The top 10 differential GO enrichment pathways in molecular functions.

Based on the cutoff criteria for differential volatile flavor compounds, nine flavor substances were upregulated, while 12 were downregulated in the 600PSE group compared with the CON group on heat maps (Figure 7C and Supplementary Table S6). Among these, the primary upregulated DVOCs included esters, hydrocarbons, and organoheterocyclic compounds, while the primary downregulated compounds consisted of ethers, aldehydes, alcohols, and benzenoids. The upregulated and downregulated flavor sensory characteristics were visualized using Sankey diagrams. Among the identified and annotated upregulated flavor compounds (Figure 7D), (E)-9-Octadecenoic acid ethyl ester, hexadecanoic acid ethyl ester, 10-Undecenoic acid ethyl ester, tetradecanoic acid ethyl ester, and octadecanoic acid ethyl ester, classified as esters, contributed to waxy, fruity, and wild sensory flavor characteristics. Downregulated pentanal (aldehyde), 1-pentanol (alcohol), 1-hexanol (alcohol), and o-xylene (benzenoid) contributed to sweet, green, and nutty sensory flavor profiles (Figure 7E).

### 3.5. Analysis and Difference of Expressed Genes in Goat Meat

Compared with the CON group, a total of 341 differentially expressed genes (DEGs) were identified in the 600PSE group (*p* < 0.05, |log_2_FC| > 1; Figure 8B,C), including 251 upregulated and 90 downregulated genes (Supplementary Table S7). Functional annotation indicated that these DEGs were primarily enriched in biological processes and molecular function categories. GO enrichment analysis revealed that the most represented terms included cellular process, biological regulation, and metabolic process in the biological process (BP) category; binding, catalytic activity, and transporter activity in the molecular function (MF) category; and cellular anatomical entity and protein-containing complex in the cellular component (CC) category (Figure 8D).

Further analysis (Figure 9) focusing on redox-related processes showed that DEGs were significantly enriched in superoxide metabolic process (*p* < 0.001, BP), reactive oxygen species metabolic process (*p* < 0.001, BP), oxidoreductase activity (*p* < 0.01, MF), and oxidoreductase activity, acting on paired donors, with incorporation or reduction of molecular oxygen, NAD(P)H as one donor and incorporation of one atom of oxygen (*p* < 0.001, MF). Specifically, in the superoxide metabolic process category, five genes were upregulated in the 600PSE group compared with the CON group. Similarly, within the reactive oxygen species metabolic process category, eight genes were upregulated, whereas only one gene was downregulated. In the oxidoreductase activity, acting on paired donors, with incorporation or reduction of molecular oxygen, NAD(P)H as one donor, and incorporation of one atom of oxygen category, there were five genes upregulated in the 600PSE group. In addition, 20 genes were upregulated in the 600PSE group, but two genes were downregulated in the oxidoreductase activity category.

### 3.6. Correlation Analysis

**Figure 10:**
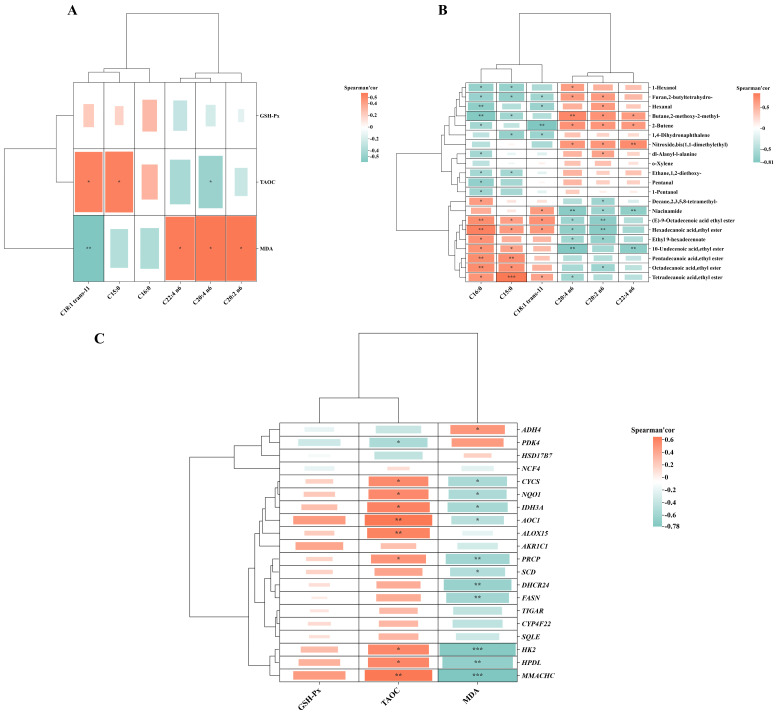
Correlation analysis between key antioxidant parameters, free fatty acids, volatile flavor compounds, and DEGs in Hainan black goats in the CON and 600PSE groups. (**A**) Correlation analysis between key antioxidant parameters and free fatty acids in Hainan black goats. (**B**) Correlation analysis between key free fatty acids and volatile flavor compounds in Hainan black goats. (**C**) Correlation analysis between key antioxidant parameters and DEGs in Hainan black goats between the CON and 600PSE groups. * *p *< 0.05, ** *p* < 0.01, and *** *p* < 0.001.

Correlation analysis revealed distinct associations among antioxidant status, free fatty acid composition, volatile flavor compounds, and antioxidant-related gene expression. Serum antioxidant parameters were differentially associated with muscle free fatty acid profiles. In general, lower MDA levels were positively associated with several long-chain n-6 polyunsaturated fatty acids, including C20:4n6, C22:4n6, and C20:2n6, whereas T-AOC showed positive correlations with C18:1 trans-11 and C15:0 (*p* < 0.05, Figure 10A).

Free fatty acid composition was further associated with the formation of volatile flavor compounds. Specifically, saturated fatty acids were negatively correlated with several aldehydes and alcohols, including hexanal, 1-hexanol, and pentanal, while showing positive associations with long-chain ester compounds (*p* < 0.05, Figure 10B). In contrast, n-6 polyunsaturated fatty acids exhibited negative correlations with ester compounds (*p* < 0.05).

Furthermore, antioxidant status was closely associated with the expression of several antioxidant and lipid metabolism-related genes. T-AOC was positively correlated with genes including *NQO1*, *AOC1*, *ALOX15*, *HK2*, *HPDL*, and *MMACHC*, whereas MDA showed negative correlations with *NQO1*, *FASN*, *DHCR24*, *HK2*, and *HPDL* (*p* < 0.05, Figure 10C).

## 4. Discussion

Heat stress is widely recognized to be associated with oxidative stress in livestock, which may contribute to alterations in animal growth and organ development. In the present study, dietary supplementation with PSE showed no adverse effects on pre-slaughter live weight or organ development in Hainan black goat kids under heat stress. Although absolute kidney weight increased, no significant changes were observed in serum urea, creatinine, or other biochemical parameters, indicating that renal function remained stable and was not impaired. More importantly, these physiological observations were accompanied by changes in systemic immune and antioxidant status. Serum interleukin profiles are widely regarded as sensitive indicators associated with immune responses [32]. A recognized signature of mitigated heat stress is a shift in the cytokine profile, specifically reduced pro-inflammatory markers (e.g., IL-1β, IL-6) [33,34,35] and elevated anti-inflammatory ones (e.g., IL-4) [36]. Consistent with the reported anti-inflammatory properties of PSE [14,37], our study revealed that PSE supplementation decreased IL-2 and IL-6, tended to reduce IL-1β and TNF-α, and elevated IL-4. In addition, concurrent improvements in T-AOC and GSH-Px, along with reduced MDA levels, further confirmed an enhanced antioxidant state in Hainan black goats by supplementation with PSE under heat stress, consistent with previous observations [19]. Since MDA is a major end-product of lipid peroxidation, its reduction may indicate decreased lipid oxidative damage [38], which could potentially contribute to maintaining cellular and metabolic stability under environmental stress conditions. The biological effects observed in the present study may be partially associated with the major bioactive constituents identified in PSE, particularly flavonoids and volatile oil compounds. Previous studies have reported that flavonoid-rich plant extracts (e.g., vitexin-rhamnoside) and plant essential oils rich in myristicin may enhance endogenous antioxidant defenses and alleviate inflammatory damage [39,40,41]. Therefore, the improved antioxidant and anti-inflammatory status observed in the present study may be partially related to the flavonoid and essential oil components present in PSE. Collectively, these findings suggest that dietary PSE supplementation may contribute to the modulation of antioxidant and immune-related responses in Hainan black goat kids under heat stress. However, the present study did not directly evaluate the contribution of specific plant-derived bioactive compounds from PSE, and further mechanistic studies are required.

Oxidative stress induced by heat stress may play an important role in lipid metabolism, particularly in the oxidative degradation of polyunsaturated fatty acids (PUFAs). Consistent with the observed improvements in systemic antioxidant capacity, in this study, PSE supplementation resulted in a reduction of n-6 PUFAs (e.g., C20:4n6, C22:4n6), accompanied by a relative increase in saturated fatty acids such as C15:0 and C16:0. Given that n-6 PUFAs are highly susceptible to lipid peroxidation [42], their reduction may partially reflect decreased oxidative degradation. This possibility is further supported by the concurrent reduction in serum MDA concentrations, suggesting a potential attenuation of lipid oxidative processes. Importantly, n-6 PUFAs are widely recognized as important precursors of volatile flavor compounds, particularly aldehydes and alcohols generated through lipid oxidation pathways [43]. In addition, the relative enrichment of saturated fatty acids may be associated with shifts toward more stable volatile profiles through altered precursor availability [44]. Therefore, the observed alterations in free fatty acid composition may potentially contribute to subsequent changes in volatile flavor compound formation in goat meat.

In agreement with the observed alterations in free fatty acid profiles, GC×GC–TOF-MS analysis revealed a marked shift in volatile flavor compounds. Interestingly, the upregulated DVOCs were mainly classified as long-chain esters. Esters formed via the esterification of free fatty acids and alcohols during thermal processing are generally associated with fruity and sweet aroma notes [45,46]. In the present study, increased levels of saturated fatty acids such as C16:0 may be related to their relative chemical stability and potential involvement in ester formation pathways [47], leading to the accumulation of long-chain esters such as octadecanoic acid ethyl ester and hexadecanoic acid ethyl ester. These volatile flavor compounds have been previously reported to contribute to fatty and waxy flavor characteristics [48]. Correlation analysis further indicated a significant positive association between C16:0 and the majority of upregulated long-chain ester compounds, suggesting a potential relationship between substrate availability and ester-related volatile profiles. In contrast, the downregulated DVOCs were mainly aldehydes, alcohols, and organoheterocyclic compounds. Aldehydes are important contributors to meat flavor due to their relatively low odor thresholds and are commonly associated with “fresh, grassy, green” notes, particularly in the case of short-chain aldehydes. In this study, the decreased concentrations of hexanal and pentanal led to a reduction in green and grassy notes [49]. Previous studies have demonstrated that hexanal and pentanal are typical volatile compounds produced by the autoxidation of arachidonic acids, which are derived from n-6 polyunsaturated fatty acids (PUFAs) [50,51]. Alcohols typically exhibit higher odor thresholds and contribute milder flavor characteristics in meat. The contents of 1-pentanol and 1-hexanol are also derived from the oxidation of linoleic acid [52], which mainly contribute to the green and herbal flavor profile [53,54]. These compounds have been reported to be related to lipid oxidation processes and subsequent metabolic conversions, including aldehyde reduction reactions [55]. Overall, the observed changes in volatile compound profiles may be associated with alterations in free fatty acid composition and lipid oxidation–related processes. However, the proposed relationships are primarily based on compositional and correlation analyses, and further mechanistic validation is required to confirm causal pathways underlying flavor formation.

To further explore the transcriptional responses associated with the observed physiological and metabolic changes, transcriptomic analysis was performed. Differentially expressed genes were found to be mainly enriched in redox-related biological processes and metabolic pathways. These findings suggest that PSE supplementation may be associated with transcriptional alterations related to intracellular redox regulation in muscle. *NQO1*, *AKR1C1* and *RTN4IP1* are genes commonly involved in the skeletal muscle antioxidant defense system. *NQO1* is a well-known Nrf2-responsive gene involved in quinone detoxification and the regulation of redox balance, and its increased expression has been widely considered to reflect enhanced cellular antioxidant responsiveness [56,57]. *AKR1C1* contributes to redox homeostasis by catalyzing the reduction and detoxification of lipid peroxidation-derived reactive aldehydes, thereby limiting oxidative damage to cellular macromolecules [58]. *RTN4IP1*, a mitochondrial NADPH-dependent oxidoreductase, plays an important role in regulating mitochondrial redox balance and protecting against mitochondrial ROS accumulation [59]. In the present study, *NQO1* expression showed a positive correlation with T-AOC and a negative correlation with MDA levels, suggesting a potential association between *NQO1* expression and systemic antioxidant status. In addition, the upregulation of *NCF4*, *TIGAR*, *SCD*, and *FASN* collectively indicates a coordinated metabolic reprogramming associated with enhanced redox homeostasis and lipids in skeletal muscle. *NCF4* is involved in NADPH oxidase complex function and may be associated with ROS-related signaling processes [60]. Concurrently, *TIGAR* has been reported to regulate the pentose phosphate pathway, which contributes to NADPH generation and cellular redox balance [61]. *SCD* and *FASN*-mediated lipid biosynthesis may contribute to membrane lipid remodeling and stabilization under oxidative conditions [62,63]. These changes may reflect an adaptive regulation of lipid metabolism in response to oxidative stress. Overall, these transcriptional changes suggest that PSE supplementation may be associated with changes in redox-related and lipid metabolism–related gene expression in skeletal muscle under heat stress conditions. However, these associations are based on transcriptomic and correlation analyses, and functional validation is required to confirm the underlying regulatory mechanisms in the future.

## 5. Conclusions

This study demonstrates that *Piper sarmentosum* Roxb. extract (PSE) enhanced antioxidant capacity and immune function and modulated lipid metabolism in Hainan black goats under heat stress conditions. Dietary PSE reduced free n-6 polyunsaturated fatty acid levels while promoting saturated fatty acids, accompanied by changes in volatile flavor profiles, including lower concentrations of certain aldehydes and alcohols and higher contents of long-chain esters. Transcriptomic evidence further indicated that PSE may activate redox-related pathways, supporting enhanced oxidative stability at the gene expression level. Collectively, these findings indicate that PSE may represent a promising plant-derived nutritional strategy for regulating oxidative status and flavor-associated metabolic traits in goats raised under tropical heat stress conditions. Nevertheless, further studies are needed to elucidate the underlying mechanisms involved and to assess its long-term efficacy in tropical livestock production.

## Figures and Tables

**Table 1:** Compositions and nutrient levels of the experimental basal diet (dry-matter basis, %).

Item ^1^	Content
King grass	50.00
Corn	34.60
Soybean meal	11.00
Wheat bran	1.50
NaCl	0.60
NaHCO_3_	0.30
Premix ^2^	2.00
Nutrient levels ^3^	
Crude protein	13.28
Ether extract	6.16
Neutral detergent fiber	44.04
Acid detergent fiber	19.05
Calcium	0.58
Phosphorus	0.18
Metabolizable energy, ME MJ/kg, DM	9.28
Gross energy, GE; MJ/kg DM	17.45

^1^ The concentrate compositions and nutrient levels of the basal diet. ^2^ The premix provided the following per kg of diet: VA, 15,000 IU; VD, 5000 IU; VE, 50 mg; Fe, 9 mg; Cu, 12.5 mg; Zn, 100 mg; Mn, 130 mg; Se, 0.3 mg; I, 1.5 mg; Co, 0.5 mg. ^3^ All nutrient levels were measured, and the metabolizable energy was calculated.

**Table 2:** Effect of dietary supplementation with PSE on the internal relative organ weight of male Hainan black goat kids (*n* = 9 per treatment group).

Items	Groups ^1^				SEM	*p*-Value		
	CON	200PSE	400PSE	600PSE		Treatment	Linear	Quadratic
Live weight (kg)	12.02	12.94	12.91	12.82	0.631	0.698	0.410	0.426
Organ weight (g)								
Heart	50.89	59.56	57.89	56.33	2.465	0.094	0.193	0.046
Liver	199.56	225.53	237.78	229.22	12.058	0.156	0.069	0.164
Spleen	15.11	15.78	17.56	17.22	0.828	0.136	0.035	0.550
Lungs	157.89	169.89	165.33	173.44	10.062	0.722	0.356	0.848
Kidneys	37.11 ^b^	42.00 ^ab^	44.00 ^a^	45.11 ^a^	1.894	0.025	0.004	0.325
Internal organ development index, g/kg of live weight								
Heart	4.26	4.66	4.52	4.45	0.554	0.540	0.612	0.251
Liver	16.68	17.40	18.64	17.99	0.673	0.219	0.095	0.312
Spleen	1.27	1.25	1.37	1.35	0.060	0.439	0.203	0.960
Lungs	13.35	13.19	12.59	13.43	1.654	0.870	0.930	0.563
Kidneys	3.11	3.28	3.45	3.57	0.151	0.171	0.028	0.851

^a,b^ Different small letter superscripts in a row indicate mean significant difference when treatment *p*-value < 0.05. ^1^ CON, 200PSE, 400PSE, and 600PSE; the four male Hainan black goat kid treatment groups fed the basal diet and supplemented with PSE at a level of 0, 200, 400, or 600 mg/kg DM/day, respectively.

## Data Availability

For the raw data, please contact the corresponding author. The raw reads of the transcriptome sequencing data of the muscle were uploaded to the NCBI SRA (accession number PRJNA1455417).

## Supplementary Materials

The following supporting information can be downloaded at: https://www.mdpi.com/article/10.3390/antiox15060721/s1, Supplementary Text S1: UHPLC-MS/MS and GC/MS Analysis of Chemical Composition of PSE; Table S1: The chemical components of *Piper sarmentosum* Roxb. extract (%) based on UPLC-LC/MS; Table S2: The main chemical components of the essential oil of PSE based on GC/MS; Table S3: Compositions and nutrient levels of the experimental basal diet; Table S4: Identification of sensory flavor characteristics among volatile flavor compounds; Table S5: ROAV analysis among volatile flavor compounds; Table S6: Differential volatile flavor compounds between CON and 600PSE groups; Table S7: Differential muscle genes between CON and 600PSE groups. References [64,65] are cited in the Supplementary Material.
