# Supplementary material for: Dietary Piper sarmentosum Roxb. Extract Improves Antioxidant Capacity, Lipid Metabolism and Flavor Formation in Male Hainan Black Goat Kids Under Heat Stress
Source: Antioxidants (Basel). 2026 Jun 5;15(6):721. doi: 10.3390/antiox15060721 (PMC13296006; doi:10.3390/antiox15060721)
Supplement: Supplementary file 1 [file antioxidants-15-00721-s001.zip › Supplementary Text S1.pdf]

UHPLC-MS/MS and GC/MS Analysis of Chemical Composition of PSE

1.1 Untargeted UHPLC–MS/MS analysis of bioactive compounds in PSE

An evenly mixed PSE sample (100 mg) was transferred into a 2 mL centrifuge tube containing 3 mm steel beads. Subsequently, 1 mL of 70% methanol was added, and the mixture was homogenized using an automatic sample rapid grinder (JXFSTPRP-48, 70 Hz, Shanghai Jingxin Industrial Development Co., Ltd., Shanghai, China) for 3 min. The samples were then subjected to low-temperature ultrasonic extraction (KQ-00DE, 40 kHz, Kun Shan Ultrasonic Instruments Co., Ltd, Kunshan, China) for 10 min. After centrifugation at 12,000 rpm for 10 min at 4 °C (Hunan Xiangyi Laboratory Instrument Development Co., Ltd, Changshai, China), the supernatant was collected and diluted 2–100-fold as required. Finally, 10 μL of internal standard solution (2-Amino-3-(2-chlorophenyl) propanoic acid, 100 μg/mL) was added, and the extract was filtered through a 0.22 μm PTFE membrane filter prior to liquid chromatography–tandem mass spectrometry (LC-MS) analysis.

An untargeted full-spectrum metabolomics analysis was performed to characterize the chemical composition of PSE using ultra-high-performance LC–MS/MS. The analysis was conducted on a Thermo Vanquish UHPLC system coupled with a Q Exactive HF mass spectrometer (Thermo Fisher Scientific, Germany), following the protocol previously described by Wang et al [1]. with minor modifications. Chromatographic separation was performed using an Agilent Zorbax Eclipse C18 column (1.8 μm × 2.1 mm × 100 mm,

Agilent Technologies Co., Ltd., Shanghai, China). The column temperature was maintained at 30 °C, and the flow rate was set at 0.3 mL/min. The mobile phases consisted of 0.1% formic acid aqueous solution (A) and acetonitrile (B). The injection volume was 2 μL, and the autosampler temperature was maintained at 4 °C. Mass spectrometric detection was performed in both positive and negative electrospray ionization modes under the following conditions: heater temperature, 325 °C; sheath gas flow rate, 45 arb; auxiliary gas flow rate, 15 arb; sweep gas flow rate, 1 arb; spray voltage, 3.5 kV; capillary temperature, 330 °C; and S-Lens RF level, 55%. Data were acquired in full-scan mode (m/z 100–1500) combined with data-dependent MS/MS scanning (dd-MS2, TopN = 10). The mass resolution was set to 120,000 for MS1 and 60,000 for MS2 analyses. High-energy collision dissociation (HCD) was used as the collision mode. Raw mass spectrometry data were processed using Compound Discoverer 3.3 software for retention time correction, peak detection, peak alignment, and feature extraction. Compound identification was performed based on MS/MS fragmentation data by searching against online databases, including Thermo mzCloud, as well as local spectral libraries such as Thermo mzVault.

1.2 GC–MS analysis of essential oil components in PSE

The essential oil composition of PSE obtained by distillation was analyzed using gas chromatography–mass spectrometry (GC–MS) with an Agilent 6890N gas chromatograph coupled to an Agilent 5975B mass spectrometer (Agilent Technologies Co., Ltd., Shanghai, China). The analytical procedures

were based on previously published methods[2] with minor modifications. Separation of volatile compounds was performed on an Agilent HP-5MS capillary column (30 m × 0.25 mm × 0.25 μm, Agilent Technologies Co., Ltd., Shanghai, China). The oven temperature program was set as follows: initial temperature at 50°C (held for 2 min), increased to 180°C at a rate of 5°C/min and held for 5 min, then further increased to 250°C at 10°C/min and held for 5 min. The injector temperature was maintained at 250°C, and the transfer line temperature was set at 280°C. Helium was used as the carrier gas at a constant flow rate of 1.0 mL/min. Mass spectrometric conditions were as follows: electron ionization (EI) source with an ion source temperature of 230°C and a quadrupole temperature of 150°C. Mass spectra were acquired in full scan mode over a mass range of m/z 40–600.

An appropriate amount of the sample was accurately weighed (± 0.01 g) into a flask, followed by the addition of 200 mL distilled water and several glass beads. After thorough mixing, the flask was connected to an essential oil extractor and reflux condenser. Water was added from the top of the condenser until the graduated section of the extractor was completely filled and excess water flowed back into the flask. The flask was heated in a water bath to gentle boiling and maintained under reflux for approximately 5 h until no further increase in essential oil volume was observed. After cooling briefly, the stopcock at the bottom of the extractor was opened slowly to release water until the upper level of the oil layer was approximately 5 mm above the 0 mark. The

apparatus was allowed to stand for at least 1 h, after which the stopcock was adjusted again until the upper surface of the oil layer was exactly aligned with the 0 mark. The obtained essential oil was transferred to GC–MS analysis.

References：

1. Wang, X.; Chang, X.; Luo, X.; Su, M.; Xu, R.; Chen, J.; Ding, Y.; Shi, Y. An Integrated Approach to Characterize Intestinal Metabolites of Four Phenylethanoid Glycosides and Intestinal Microbe-Mediated Antioxidant Activity Evaluation In Vitro Using UHPLC-Q-Exactive High-Resolution Mass Spectrometry and a 1,1-Diphenyl-2-picrylhydrazyl-Based Assay. *Frontiers in Pharmacology* **2019**, *Volume 10 - 2019*, doi:10.3389/fphar.2019.00826.

2. Yaqub, G.; Hamid, A.; Khan, N.; Ashfaq, S.; Banzir, A.; Javed, T. Biomonitoring of workers exposed to volatile organic compounds associated with different occupations by headspace GC-FID. *Journal of Chemistry* **2020**, *2020*, 6956402.
